# Supplementary material for: A comparative analysis of co-contraction indices using synthetic EMG data: Implications for selection and interpretation
Source: PLoS One. 2026 May 29;21(5):e0343081. doi: 10.1371/journal.pone.0343081 (PMC13221005; doi:10.1371/journal.pone.0343081)
Supplement: Attachment — Submitted filename: CCI paper response to reviewers.docx [file pone.0343081.s001.docx]

**Manuscript PONE-D-26-05502**

**Response to Reviewers**

Dear Dr. Tigrini,

Thank you for the opportunity to address reviewer and editorial comments. We have provided the point-by-point response below. All tracked changes are noted as red underlined text for additions/changes, and red strikethrough text for deletions.

**Editor’s Comments to Authors:**

1. Please ensure that your manuscript meets PLOS ONE's style requirements, including those for file naming. The PLOS ONE style templates can be found at
[https://journals.plos.org/plosone/s/file?id=wjVg/PLOSOne_formatting_sample_main_body.pdf](https://nam04.safelinks.protection.outlook.com/?url=https%3A%2F%2Ftrack.editorialmanager.com%2FCL0%2Fhttps%3A%252F%252Fjournals.plos.org%252Fplosone%252Fs%252Ffile%253Fid%3DwjVg%252FPLOSOne_formatting_sample_main_body.pdf%2F1%2F010f019db8248f1c-1b5f88cc-c573-4a6e-803a-88d08f97df6a-000000%2FqLSDFXs0Ri9e4Cknz8evjDRusZe3lQq6zlpSreFZ8AA%3D258&data=05%7C02%7Chdcarey%40uic.edu%7C437978aadd984575768b08dea0e128ac%7Ce202cd477a564baa99e3e3b71a7c77dd%7C0%7C0%7C639125086082826979%7CUnknown%7CTWFpbGZsb3d8eyJFbXB0eU1hcGkiOnRydWUsIlYiOiIwLjAuMDAwMCIsIlAiOiJXaW4zMiIsIkFOIjoiTWFpbCIsIldUIjoyfQ%3D%3D%7C0%7C%7C%7C&sdata=uNu7qXs7qFknTx80pirftCfnHlfhK%2F5cwna6Supvxbo%3D&reserved=0) and <https://journals.plos.org/plosone/s/file?id=ba62/PLOSOne_formatting_sample_title_authors_affiliations.pdf>

*Response: We have reviewed the style template and revised the manuscript as appropriate.*

2. Please note that PLOS One has specific guidelines on code sharing for submissions in which author-generated code underpins the findings in the manuscript. In these cases, we expect all author-generated code to be made available without restrictions upon publication of the work. Please review our guidelines at <https://journals.plos.org/plosone/s/materials-and-software-sharing#loc-sharing-code> and ensure that your code is shared in a way that follows best practice and facilitates reproducibility and reuse.

*Response: We have made all code available without restriction on GitHub, archived with Zenodo.*

3. Please provide a complete Data Availability Statement in the submission form, ensuring you include all necessary access information or a reason for why you are unable to make your data freely accessible. If your research concerns only data provided within your submission, please write "All data are in the manuscript and/or supporting information files" as your Data Availability Statement.

*Response: All data are in the manuscript and/or supporting information files.*

4. We note that the grant information you provided in the ‘Funding Information’ and ‘Financial Disclosure’ sections do not match. When you resubmit, please ensure that you provide the correct grant numbers for the awards you received for your study in the ‘Funding Information’ section.

*Response: We have revised the grant information to match in the ‘Funding Information’ and ‘Financial Disclosure’ sections. The correct grant number is included in the ‘Funding Information’ section.*

5. Thank you for stating the following financial disclosure:
The authors disclosed receipt of the following financial support for the research, authorship, and/or publication of this article: Research reported in this publication was supported by the Department of Defense under Award No. HT9425-24-1-0212. The content is solely the responsibility of the authors and does not necessarily represent the official views of the Department of Defense.

Please state what role the funders took in the study. If the funders had no role, please state: "The funders had no role in study design, data collection and analysis, decision to publish, or preparation of the manuscript." If this statement is not correct you must amend it as needed. Please include this amended Role of Funder statement in your cover letter; we will change the online submission form on your behalf.

*Response: Thank you. The funder had not role. We have included the requested statement in our cover letter.*

6. If the reviewer comments include a recommendation to cite specific previously published works, please review and evaluate these publications to determine whether they are relevant and should be cited. There is no requirement to cite these works unless the editor has indicated otherwise.

*Response: N/A*

7. Please review your reference list to ensure that it is complete and correct. If you have cited papers that have been retracted, please include the rationale for doing so in the manuscript text, or remove these references and replace them with relevant current references. Any changes to the reference list should be mentioned in the rebuttal letter that accompanies your revised manuscript. If you need to cite a retracted article, indicate the article’s retracted status in the References list and also include a citation and full reference for the retraction notice.

*Response: We have checked the reference list, which is complete. We have updated the formatting for consistency between items (i.e., abbreviation of journals)*. *We have also added two references regarding the color palettes used in manuscript figures, reproduced below:*

62. Crameri F. Scientific colour maps. Zenodo; 2023. doi:10.5281/zenodo.8409685

63. Crameri F, Shephard GE, Heron PJ. The misuse of colour in science communication. Nat Commun. 2020;11: 5444. doi:10.1038/s41467-020-19160-7

**Additional Editor Comments**:

The manuscript presents a well-written and comprehensive review of muscle co-contraction indexes. However, please update it by addressing all the concerns raised by the reviewers.

*Response: Thank you for your feedback. We have included our response to the reviewer below.*

**Reviewer’s Comments to Authors:**

Reviewer 1

The manuscript is well written and the methodological framework is carefully developed. The comparison of multiple co-contraction indices is particularly interesting and represents a valuable methodological contribution. I only have a minor suggestion regarding Figure 9. While the normalization parameters are mentioned in the manuscript and the script used to generate the synthetic EMG signal is available in the GitHub repository, the intermediate steps that lead from those parameters to the plot shown in Figure 9 are not entirely clear from the text. It would therefore be helpful if the authors could briefly clarify the processing steps that transform the reported normalization parameters into the final signal representation shown in the figure.

*Response: Thank you for your review. There are no processing steps as this is synthetic EMG. We simply used 0.6, 1.0, and 1.4 as the maximum height of a linear function (with zero intercept) to create the dotted, dash-dotted, and dashed lines in Figure 9. Bar plots on the left represent the co-contraction index calculated from the solid line vs each dash/dot line. We have updated the caption of figure 9 (excerpt below) to clarify.*

Revised Text: **Figure 9**. Amplitude normalization influences each CCI in distinct ways. In panels A and B, the amplitude of EMG_1_ (solid black lines) is fixed, while that of EMG_2_ varies to represent three amplitude normalization conditions: non-normalized (dotted line, maximum amplitude 1.4), within-task normalization (dash-dotted line, maximum amplitude 1), and MVC normalization (dashed line, maximum amplitude 0.6). Panels C and D show bar plots of co-contraction values for all six CCIs, calculated from the three EMG_1_ and EMG_2_ combinations in panels A and B. Amplitude-driven CCIs (i.e., Rudolph and Unnithan-Frost) are most affected when amplitude normalization reduces *both* EMG_low_ and total EMG (panel C). In contrast, shaped-based CCIs, like Falconer-Winter, are most sensitive when normalization alters *only one* *of* EMG_low_ and total EMG (panel D). Abbreviations: CCI_SR_: simple ratio; CCI_FW_: Falconer-Winter; CCI_TS_: Thoroughman-Shadmehr; CCI_R_: Rudolph; CCI_UF_: Unnithan-Frost; CCI_T_: temporal.
